# RNA-seq analysis reveals key genes associated with downregulation of APE1 in esophageal squamous cell carcinoma

**DOI:** 10.3389/fgene.2025.1549371

**Published:** 2025-04-22

**Authors:** Alan Chu, Xiao Liu, Shijia Liu, Mengxi Li, Rui Song, Lanlan Gan, Yongtai Wang, Zongwen Liu, Chen Sun

**Affiliations:** Department of Radiation Oncology, The Second Affiliated Hospital of Zhengzhou University, Henan, China

**Keywords:** RNA-seq, Ape1, esophageal cancer, molecular target, bioinformatics

## Abstract

**Objective:**

This study aims to explore the impact of APE1 gene knockout on the transcriptome of esophageal cancer cells and conduct a preliminary screening of APE1-regulated target genes to provide a basis for understanding APE1 target genes and finding new anti-esophageal cancer therapeutic targets.

**Methods:**

We collected 100 patients with esophageal squamous cell carcinoma (ESCC), analyzed the expression of APE1 in ESCC by immunohistochemical, and analyzed the overall survival. TE-1 cells with APE1 knockout were used for transcriptome sequencing (RNA sequencing, RNA-Seq) detection, and GO and KEGG enrichment analysis of differentially expressed genes was conducted. Protein-protein interaction (PPI) network analysis was performed on the genes in the intersection of differential genes between the two sequencing datasets. The qRT-PCR and Western blotting experiments were employed to confirm the effect of APE1 knockdown on the expression levels of FN1, TNF, and IL-6 in esophageal cancer cells.

**Results:**

APE1 highly expressed in ESCC tissue, and its high expression leads the worse OS. The stable transfected TE-1 cell line with knockdown of the APE1 gene was successfully constructed, with a knockdown efficiency of 100%. RNA-seq analysis revealed that 2,060 differential genes were detected between APE1-KO stably transfected cells and their corresponding APE1-YD cells, with 1,063 upregulated genes and 997 downregulated genes. RNA-seq analysis found that differentially expressed genes after APE1 knockout in TE-1 cells were primarily enriched in pathways related to metabolism, extracellular matrix, inflammatory response, and angiogenesis. PPI network analysis demonstrated that FN1, TNF, and IL-6 may be essential target genes of APE1. The three core genes of FN1, TNF, and IL-6 were confirmed using qRT-PCR and Western blotting, and the results were consistent with the transcriptome sequencing results.

**Conclusion:**

Knocking out APE1 can affect the function, related pathways, and downstream target gene expression of ESCC cells. APE1 can promote the transcriptional expressions of FN1 and IL6 genes while inhibiting the TNF gene. FN1, TNF, and IL-6 may be potential target genes regulated by APE1 in esophageal cancer.

## 1 Introduction

In 2020, esophageal cancer had the eighth-highest incidence rate of any malignant tumor and the sixth-highest mortality rate globally. Approximately 600,000 patients were newly diagnosed with esophageal cancer (Sung et al., 2021). Despite significant advances in medical technology and treatment strategies in recent years, the prognosis for patients with esophageal cancer remains unsatisfactory, with a low 5-year survival rate. Moreover, the pathogenesis and therapeutic targets of this disease remain unclear (Zhu et al., 2023). Consequently, an in-depth study of the molecular mechanisms of esophageal cancer is critical for improving its early diagnosis and treatment effects.

Apurinic/apyrimidinic (AP) endonuclease (apurinic/apyrimidinic endodeoxyribonuclease, APE) is an enzyme involved in the DNA base excision repair (BER) pathway. It repairs damaged or mismatched DNA and plays a major role in nucleotides (Malfatti et al., 2021). Humans have two AP endonucleases: apurinic/apyrimidinic endodeoxyribonuclease 1 (APE1) and apurinic/apyrimidinic endodeoxyribonuclease 2 (APE2). APE1 has AP endonuclease, 3′-5′ exonuclease (Bakman et al., 2022), 3-phosphodiesterase, 3′RNA phosphatase, and 3′-ribonuclease activities (Gros et al., 2004). APE1 provides most AP endonuclease activity in cells; accordingly, it is regarded as the primary AP endonuclease in human cells. Previous studies demonstrated that APE1 has two main functions: first, it can repair oxidative and alkylation damage in the BER pathway (Zhang and Wang, 2010); second, as a redox signaling protein, it regulates certain transcription factors (STAT3, HIF-1α, and NFκB) activity (Zhang and Wang, 2010; Logsdon et al., 2016; Ando et al., 2008; Caston et al., 2021). This is because it is the critical link between DNA repair and redox, which plays a vital role in tumor occurrence and development. APE1 plays a role in cancer development and chemotherapy resistance due to its abnormal expression and subcellular localization in various cancers. Hence, APE1 is an emerging therapeutic target for treating multiple cancers (Caston et al., 2021).

APE1 overexpression can be detected in various malignant tumors, such as non-small-cell lung cancer, prostate cancer, ovarian cancer, cervical cancer, malignant peripheral nerve sheath tumors, liver cancer, and colon cancer (Long et al., 2021; McIlwain et al., 2018; Wen et al., 2016; Li et al., 2021; Gampala et al., 2021; Lu et al., 2021; Hong et al., 2023). Prior research revealed that in esophageal cancer, elevated APE1 can lead to homologous recombination and cell cycle dysregulation, resulting in genome instability, tumorigenesis, and chemotherapy resistance (Lu et al., 2023). The study of Kumar et al. demonstrate that APE1 overexpression in esophageal adenocarcinoma models disrupts HR repair fidelity and cell cycle checkpoint regulatory networks, synergistically inducing chemoresistance and tumor clonal evolution (Kumar et al., 2023). Aberrant overexpression of APE1 protein promotes oncogenesis through a dual mechanistic framework: First, dysregulation of its endonuclease activity induces DNA repair pathway perturbation, particularly triggering error-prone homologous recombination (HR) repair programs under replication stress conditions. This fidelity-compromised HR repair mode significantly increases the burden of oncogenic mutations and the frequency of chromosomal rearrangements, thereby driving cascading amplification of genomic instability (Bhat et al., 2018). Second, the redox regulatory function of APE1 activates pro-survival transcription factor complexes such as NF-κB and HIF-1α through targeted modulation, constructing a pro-tumorigenic microenvironment (Sriramajayam et al., 2021).

However, research on the effects of APE1 knockdown on ESCC cell development and function is limited, and the key genes and biomarkers involved in its pathway and function are still unclear. In this study, we validated the expression of APE1 in ESCC tissues and its impact on overall survival. Then the APE1-KO and APE1-YD ESCC cell lines were constructed. RNA-seq technology was used to sequence the transcriptomes of the two cell groups to obtain comprehensive gene expression information. Genes that are significantly differentially expressed in the stably transfected cell lines with APE1 knockout were identified by comparing the gene expression profiles of the two groups of samples. Then, bioinformatics analysis was performed on the differentially expressed genes (DEGs), including gene function annotation, pathway enrichment analysis, and PPI network construction. These analyses will help us understand the potential functions and biological processes involved in APE1 in ESCC cells. Finally, we will verify the expression levels of DEGs through quantitative real-time polymerase chain reaction (qRT-PCR) and further confirm the results of RNA-seq analysis using Western blotting analysis to detect changes in protein levels.

This study aims to investigate the expression of differentially expressed genes in APE1-KO in ESCC cells and explore their potential functions, providing new insights and targets for the pathogenesis and treatment of esophageal cancer. Further research may help develop therapeutic strategies targeting APE1-KO and lay the necessary foundation for personalized cancer treatment.

## 2 Materials and methods

### 2.1 Patients

Collect ESCC patients hospitalized at the Second Affiliated Hospital of Zhengzhou University from January 2016 to December 2020, and screen patients based on inclusion and exclusion criteria. The cutoff time for our survival study was set to 31 December 2023. A total of 100 patients (all surgical patients) were screened. All samples were confirmed by pathological examination according to the American Joint Committee on Cancer (AJCC) Cancer Staging Manual (8th edition). The inclusion criteria are: 1. There is complete case data in our hospital, and the pathological diagnosis is ESCC; 2. Age range from 18 to 75 years old; 3. Expected survival >3 months. Exclusion criteria: 1. Patients who have previously undergone other anti-tumor treatments (including chemotherapy, targeted therapy, immunotherapy, or radiotherapy); 2. Patients with other malignant tumors or serious diseases that affect treatment effectiveness or survival; 3. According to the Eastern Cooperative Oncology Group (ECOG) scale, Patients with Performance Status (PS) score >2. All patients have informed consent.

This study was performed in line with the principles of the Declaration of Helsinki. Approval was granted by the Ethics Committee of The Second Affiliated Hospital of Zhengzhou University (number: 2022287).

### 2.2 Scoring criteria for APE1 expression in immunohistochemistry

Select five different areas of tissue slices using a high-power field of view (400×), with at least 100 cells in each field counted. Positive results are determined based on the appearance of brown yellow or brown APE1 in nuclear, cytoplasmic, or stromal cell staining. Tumor cell positivity score: 0 points for 0%–5%, 1 point for 6%–25%, 2 points for 26%–50%, 3 points for 51%–75%, and 4 points for 76%–100%. Positive cell staining intensity score: negative score is 0 points, weak positive score is 1 point, moderate positive score is 2 points, and strong positive score is 3 points. The final score is the tumor cell positivity rate multiplied by the strength of positive cell staining. A score of 0 is (−), 1–4 is (+), 5–8 is (++), and 9–12 is (+++). (−), (+), and (++) indicate low expression of APE1, while (+++) indicates high expression of APE1. Two pathologists simultaneously read and judge the slices.

### 2.3 Construction of APE1 knockout cell lines

TE-1 cells line was obtained from Procell (Wuhan, Hubei, China). The cell line has been identified by Short Tandem Repeat (STR). The construction of APE1 knockout cell lines were completed by Sangon Biotech (Shanghai, China). Electroporation was used to knock out the target gene of the target cell using the principle of CRISPR/Cas9 gene editing technology, after which the individual cells are isolated and cultured for monoclonalization. Following amplification and culture, PCR and sequencing are used to confirm the target gene knockout, yielding a positive result for the monoclonal target gene knockout. Using APEX1-210 as the transcript, a target was created for exons 3 and 5 (the guide RNA with less off-target as the target was selected). The gRNA sequence is as follows:

gRNA-A1: GTT​GGG​TCT​ATA​GTT​AAC​GC CGG

gRNA-A2: GTT​GAG​GGG​GCT​TAT​TTC​CC AGG

The clone sample genome was extracted for PCR detection to identify the gRNA targeting site and knockout band. The successful knockout of the monoclonal cell line was preliminarily determined and sequenced. The sequencing results were compared using SnapGene software to determine the successful knockout of the monoclonal cell line.

### 2.4 RNA-seq library preparation and sequencing

The APE1-KO (APE1 knockout) and APE1-YD (negative control) cell lines were generated through CRISPR/Cas9 gene editing, and the knockout efficiency was validated by comparing RT qPCR and Snapgene software. Each group processed three biological replicates (a total of six samples) for RNA extraction. Using TRIzol^®^ Total RNA was isolated using a reagent (Invitrogen, 15596-026) and then subjected to DNase I (RNase free, Thermo Fisher, EN0521) treatment to eliminate genomic DNA contamination. RNA integrity (RIN ≥ 8.0) and quantity (≥1 μg) were evaluated using Fragment Analyzer.

The transcriptome library preparation used the DNBSEQ-T7 platform (MGI Tech). First, RNA is extracted using MGI’s MGIEasy RNA Extraction Kit and processed on automated systems like the MGISP-100/960 workstation, followed by rRNA depletion (MGIEasy rRNA Removal Kit) or mRNA enrichment. Next, directional cDNA libraries are constructed via the MGIEasy RNA Directional Library Prep Kit, which includes fragmentation, reverse transcription, end repair, A-tailing, adapter ligation, and PCR amplification. For DNB (DNA Nanoball) generation, linear DNA is circularized using the MGI DNB Preparation Kit on the MGIDL-T7 instrument, followed by rolling circle amplification to produce high-fidelity DNA nanoballs. Finally, DNBs are loaded onto patterned flow cells and sequenced on the DNBSEQ-T7 platform using the DNBSEQ-T7 High-Throughput Sequencing Reagent Kit V3.0, enabling paired-end reads (PE100/150) with Q30 ≥85% and throughput up to 1.5 Tb per run.

### 2.5 Bioinformatics and data analyses

#### 2.5.1 Quantification of gene expression

Gene expression quantification was performed using StringTie v2.1.7 to assemble transcripts from aligned reads, with the human reference genome (GRCh38) and GENCODE v41 annotations. RSEM v1.3.3 was then used to estimate gene and transcript abundances, with Bowtie2 v2.3.5 alignments and default parameters. FPKM values were calculated to normalize for gene length and sequencing depth. Principal component analysis (PCA) was conducted using the prcomp function in R on log_2_(FPKM + 1)-transformed data to assess sample clustering and batch effects.

#### 2.5.2 PCA analysis of genes

Principal component analysis (PCA) attempts to recombine many original indicators with certain correlations into a new set of independent comprehensive indicators to replace the original ones. PCA uses linear algebra calculation methods to perform dimensionality reduction and principal component extraction on tens of thousands of genetic variables to evaluate differences between groups and sample duplication within groups. PCA analysis was conducted on all samples’ gene expression values (expressed as FPKM) to determine the main components and then draw a PCA plot.

#### 2.5.3 Differential expression analysis

To screen DEGs, the expected count in the gene quantification results was used as input data for differential expression analysis. The analysis software was the R language package DESeq2. The screening threshold was FDR <0.05, log FC (fold change (condition 2/condition 1) for a gene) >1, or log FC < −1. After the DEGs were detected, we visualized DEGs using volcano plots (ggplot2,v3.4.3) and cluster plots (pheatmap, v1.0.12).

The used gene ontology (GO) enrichment analysis method was hypergeometric distribution, and GO terms with FDR≤0.05 were employed as significantly enriched GO terms. The top 20 most significantly enriched pathways were selected, and a GO enrichment scatter plot of DEGs was drawn. The Kyoto encyclopedia of genes and genomes (KEGG) is the primary public database for pathways (Kanehisa, 2008). Pathway significant enrichment analysis uses the KEGG pathway as the unit and the hypergeometric test to identify pathways significantly enriched in DEGs compared to the entire genome. Pathways with FDR≤0.05 are defined as pathways that are significantly enriched in DEGs. R software was used in conjunction with self-written scripts, and FDR was calculated using the Benjamini–Hochberg method (referred to as the BH method) for pathway enrichment analysis. The top 20 most significantly enriched pathways were selected, and a KEGG enrichment scatter plot of DEGs was drawn. The GO enrichment and KEGG enrichment degrees were measured using the Rich factor, Q value, and the number of genes enriched in this pathway, respectively. The Rich factor refers to the ratio of the number of differential genes enriched in the pathway to the number of annotated genes—the more significant the Rich factor, the greater the degree of enrichment. The Q value is the P value after multiple hypothesis testing corrections. The value range of the Q value is [0,1]; the closer it is to zero, the more significant the enrichment is. Afterwards, pathview (v1.38.0) was used to integrate expression profile data and visualize KEGG pathways.

#### 2.5.4 Protein interaction analysis

The STRING protein interaction database (http://string-db.org/) records protein cooperation relationships of many species. Using this database, PPI networks can be constructed. Diamond software was used to align the sequences in the differential gene set to the protein sequences of the reference species in the STRING database, and the aligned protein interaction relationships of the reference species were used to construct an interaction network.

The resulting file provides a differential gene-protein interaction network data file, which can be directly imported into Cytoscape software for visual editing. The download path of Cytoscape software is https://cytoscape.org/download.html. CytoHubba was applied in the software to predict essential disease genes, and MCC, MNC, and Degree scores were chosen as prediction methods.

### 2.6 qRT-PCR

The cells were washed twice with prechilled phosphate-buffered saline (PBS) (Invitrogen). Then, 1 mL of Trizol cell lysis solution (Invitrogen) was added to each well, total RNA was extracted from the cells according to the instructions, and the purity and concentration of the RNA were detected. According to the instructions, RNA was transcribed into cDNA, and qRT-PCR was performed. Moreover, 2 μL of reverse-transcribed cDNA served as the reaction template, and a mixed reaction system was prepared with 10 μL of 2 × SYBR Green 480 reaction solution (Vazyme, Nanjing), 1 μL of upstream and downstream primers, and 6 μL of ddH2O. A real-time fluorescence quantitative PCR instrument (Bio-Rad, CFX Connect) was used for the following cycle: predenaturation at 95°C for 10 min; denaturation at 95°C for 30 s; annealing at 60°C for 20 s; extension at 72°C. Forty cycles were the reaction conditions. The relative expression of genes was calculated using the 2^−ΔΔCT^ method with GAPDH as the internal reference. The quantitative primers involved are listed in Table 1.

**TABLE 1:** Primer information list.

Primer	Sequence
GAPDH	F: GGA​AGC​TTG​TCA​TCA​ATG​GAA​ATC
	R: TGA​TGA​CCC​TTT​TGG​CTC​CC
FN1	F: GCA​TTG​CCA​ACC​TTT​ACA​GAC​C
	R: TTG​GAA​ATG​TGA​GAT​GGC​TGT​G
TNF-α	F: GCT​GCA​CTT​TGG​AGT​GAT​CG
	R: ATG​AGG​TAC​AGG​CCC​TCT​GA
IL-6	F: TTC​GGT​CCA​GTT​GCC​TTC​TC
	R: AGT​GCC​TCT​TTG​CTG​CTT​TCA

### 2.7 Western blotting

To detect the expression level of total protein, 200 μL of cell protein lysis buffer (RIPA: PMSF = 100:1) was added to each well of a six-well cell culture plate. Total protein was extracted from cells according to the instructions, and the concentration of each group of proteins was detected. Equal and appropriate amounts of protein samples were collected, and sodium dodecyl sulfate-polyacrylamide gel electrophoresis (SDS-PAGE) (MeilunBio, Dalian)was performed. After the electrophoresis, the proteins were transferred from the gel to a PVDF membrane, and then the membrane was placed on a 5% skim milk powder medium, blocked at room temperature for 2 h. After blocking, antibody hybridization tape was used to incubate APE1 and β-tubulin antibodies (diluted with TBST at a ratio of 1:1,000) and incubated overnight at 4°C. The next day, the membrane was washed three times with TBST buffer, 10 min each time; then, hybridization was used for incubation with the secondary antibody of the corresponding species (diluted with TBST at a ratio of 1:1,000), followed by incubation at room temperature for 2 h. Afterward, the membrane was washed three times with TBST, 10 min each time. Finally, the chemiluminescence chromogenic solution was added dropwise and put into the chemiluminescence instrument; the relevant parameters were set according to the instrument’s operating procedures, and pictures were taken and saved to analyze the data.

### 2.8 Statistical analysis

Nominal variables were analyzed using the Chi-square. The OS was calculated from the time of diagnosis of esophageal cancer until death from any cause or final follow-up. Median OS with 95% confidenceintervals (CI), were calculated via the Kaplan-Meier method andcompared with the log-rank test. The statistical analysis involved in this study was conducted using GraphPad Prism version 9 software. All validation experiments were performed independently at least three times. Differences between the two groups were analyzed using an independent samples t-test. Quantitative data were expressed using the mean ± standard deviation method. P < 0.05 was considered to be statistically different.

## 3 Results

### 3.1 Expression of APE1 in ESCC tissue

**FIGURE 1:**
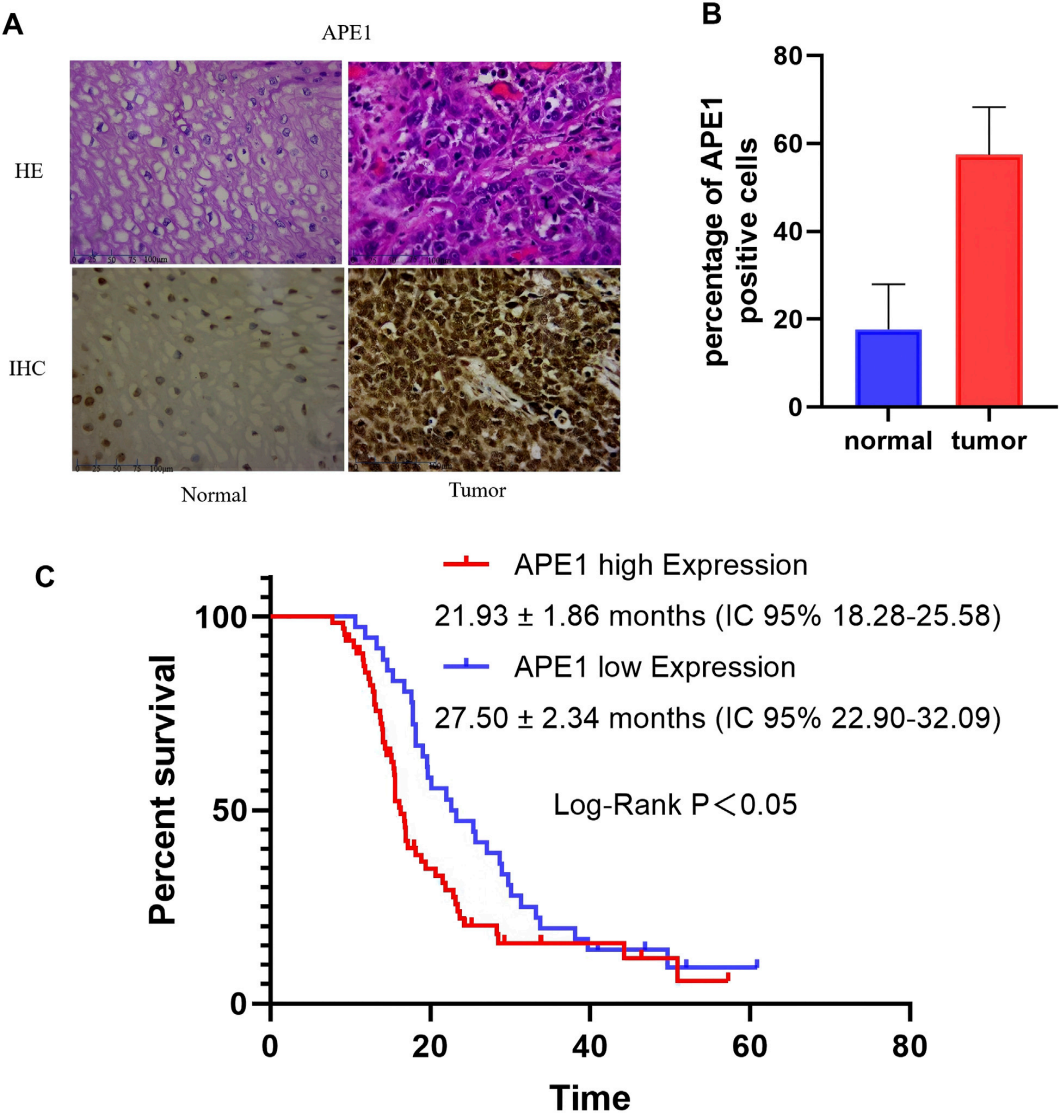
The expression of APE1 in ESCC tissue and its impact on overall survival. **(A)** Immunohistochemical staining determination of APE1 levels in normal tissue and ESCC tissue (40×); **(B)** The percentage of APE1 positve cells in normal tissue and ESCC tissue; **(C)** Relationship between APE1 expression and overall survival of patients.

We collected 100 ESCC patients treated in the Second Affiliated Hospital of Zhengzhou University. The baseline characteristics of patients shown in Table 2. The analysis results showed that the expression level of APE1 in ESCC tissue was correlated with TNM staging, lymph node metastasis, and depth of invasion, but not significantly correlated with gender, age, and tumor differentiation. Immunohistochemical detection of APE1 expression in ESCC tissue and adjacent normal tissues. The percentage of APE1 positive cells in ESCC tumor tissues was significantly higher than that in adjacent tissues (P < 0.05) (Figures 1A, B).

**TABLE 2:** Baseline characteristics of patients.

	No.	APE1expression		χ^2^	P value
		Low expression (n = 43)	High expression (n = 57)		
Age (y)					
≤60	35	14	21	0.20	0.66
>60	65	29	36		
Gender					
Male	60	24	36	0.55	0.46
Female	40	19	21		
TNM stage					
I + II	47	28	19	9.94	<0.05
III	53	15	38		
Lymph node metastasis					
Yes	56	17	39	8.30	<0.05
No	44	26	18		
Differentiation					
Well	6	5	1	1.85	0.17
Moderately	62	28	34		
Poorly	22	10	22		
Invasion depth					
Mucosa	10	9	1	8.75	<0.05
Muscularis	56	19	37		
Serosa	24	15	19		

We collected overall survival data from 100 patients from 2016 to 2023. The Kaplan Meier survival curve was used to analyze the relationship between the expression of APE1 and the overall survival period of the patients. According to the scoring criteria for APE1 expression, patients were divided into APE1 high expression and APE1 low expression groups. The median OS in the high expression group was 21.93 ± 1.86 months (IC 95% 18.28–25.58), while the median OS in the low expression group was 27.50 ± 2.34 months (IC 95% 22.90–32.09), P < 0.05 (Figure 1C).

### 3.2 Construction of stably transfected cell lines with APE1 gene knockout

**FIGURE 2:**
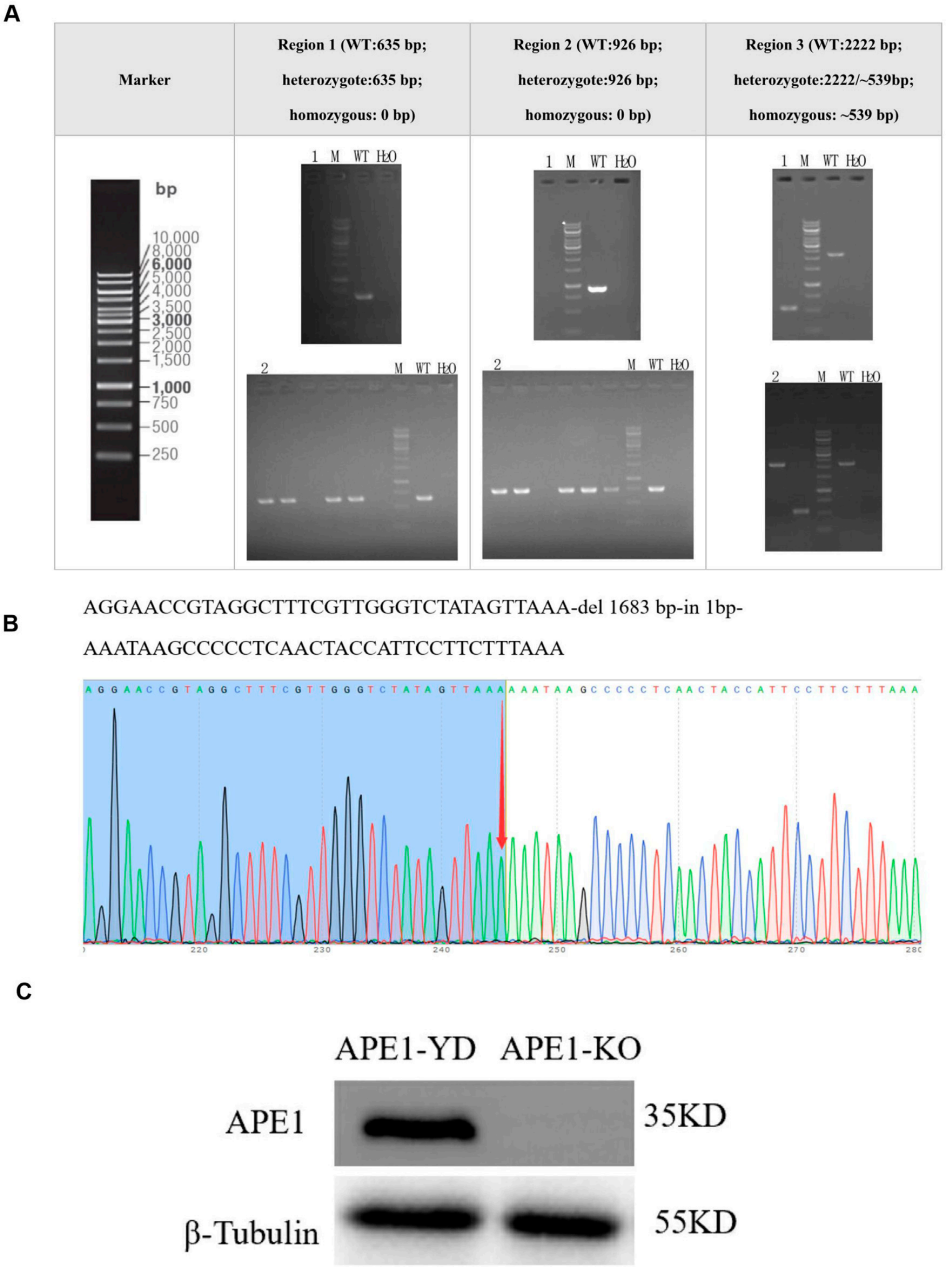
Construction of stably transfected cell lines with APE1 gene knockout. **(A)** PCR identification results: 1 represents sample 1, 2 represents sample 2, M was the Marker, WT was the negative control group, and H_2_O represented the blank control group; **(B)** Sequencing results of Sample 1. The arrow points to the homozygous mutation site; **(C)** Western blotting detection results of APE1 in two types of cells.

Sample 1 was a homozygous clone identified by PCR, and Sample 2 was a negative single clone identified by PCR. Figure 2A demonstrates no PCR amplification of the APE1 gene in Well 1 of the strip. Sample 1 was a successfully constructed stably transfected cell line with the knockout of the APE1 gene; there was PCR amplification of the APE1 gene in Well 2 of the strip. PCR amplification Sample 2 was the stably transfected cell line of the constructed negative control. From the sequencing results in Figure 2B, it is observed that Sample 1 was homozygous, and the knockout efficiency was 100%. The Western blotting diagram of APE1 protein in the APE1-KO and APE1-YD cell lines revealed the expression of APE1 protein in the APE1-YD cell line, and the total expression of the APE1-KO cell line exhibited no expression of APE1 protein (Figure 2C). The above three identification methods all indicate that a stably transfected cell line with APE1 gene knockout was successfully constructed.

### 3.3 Differential gene screening and clustering heatmap

**FIGURE 3:**
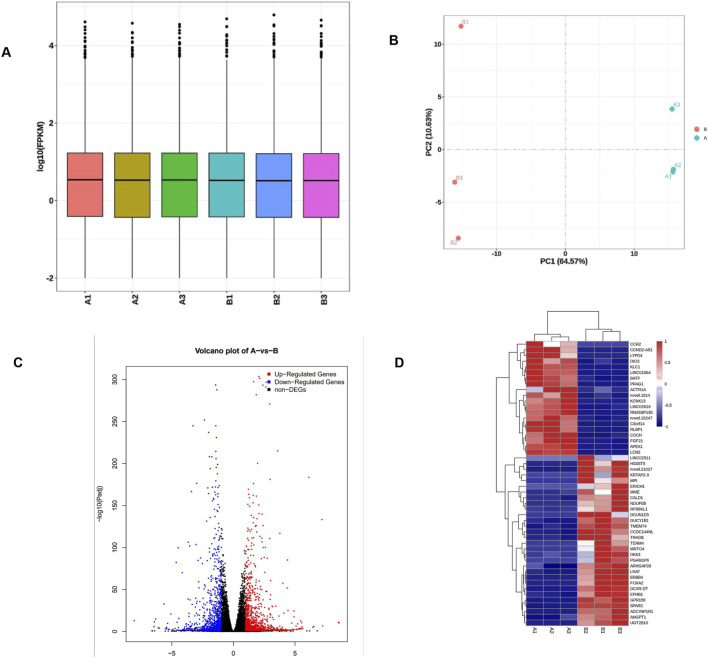
Quality control of APE1 knockout cells and clustering analysis of differentially expressed genes. **(A)** The abscissa of the FPKM box plot is divided into the negative control group (A1/A2/A3) and the knockout group (B1/B2/B3). The ordinate is the log_10_ value of FPKM; **(B)** The first principal component of the PCA diagram (PC1) is used as the X-axis, and the second principal component (PC2) as the Y-axis. The PC1 value is 64.57%, meaning that the difference in the X-axis can explain 64.57% of the comprehensive analysis results; the PC2 value is 10.63%, meaning that the difference in the Y-axis can explain 64.57% of the comprehensive analysis results; **(C)** The volcano plot of DEGs demonstrates genes with non-significant differences in gray and genes with significant differences in red (upregulated) and blue (downregulated); the X-axis is the display of log_2_ fold change, and the Y-axis direction is −log_10_ P display of value; **(D)** Cluster analysis of differential genes. The abscissa of the heatmap is the sample name, and the ordinate is the FPKM normalized value of the differential genes. The redder the color, the higher the expression level; the bluer, the lower the expression level.

In the early stage of this study, CRISPR/Cas9 gene editing technology was used to construct a TE-1 cell line with stable knockout of the APE1 gene, named B1/B2/B3 cell line, while its control cell line was A1/A2/A3 cell line. RNA-seq detection was performed on A1/A2/A3 and B1/B2/B3 cells, respectively, to explore the impact of knocking out APE1 on the transcriptome of ESCC cells. The FPKM box plot (Figure 3A) revealed that the log_10_ (FPKM) of 50% of the genes in the two groups is concentrated between greater than −1 and 1, indicating that the standardized FPKM values between the six samples in the two groups are relatively balanced, meaning that the distribution of RNA expression is reasonable, uniform, and stable, and difference analysis can be performed. The PCA diagram (Figure 3B) illustrates that the consistency within the two groups of samples is good, and the differences between the groups are obvious. A total of 2,060 DEGs were screened out based on the sequencing results, including 1,063 DEGs with upregulated expression and 997 DEGs with downregulated expression. A volcano plot for each group of sample comparisons (Figure 3C) and a cluster analysis heatmap of the top 50 most significant DEGs (Figure 3D) were drawn to display the DEGs visually.

### 3.4 Differential gene analysis

**FIGURE 4:**
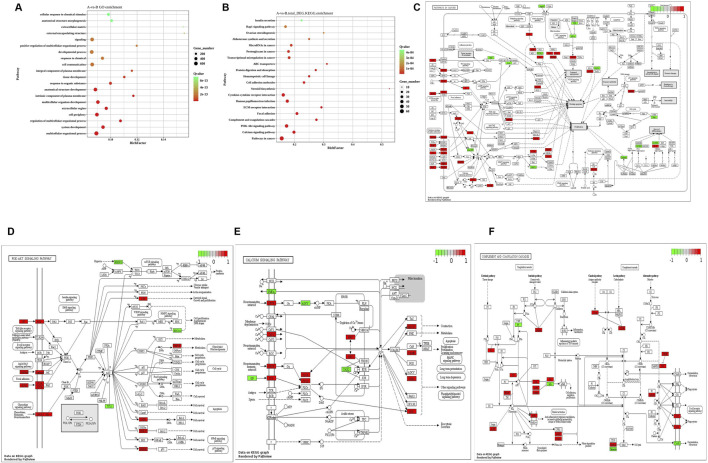
GO analysis and KEGG analysis scatter plot of differential genes. **(A)** GO analysis scatter plot of differential genes between two groups of samples; **(B)** KEGG analysis scatter plot of differential genes between two groups of samples; **(C)** Pathways in cancer. **(D)** Calcium signaling pathway; **(E)** PI3K−Akt signaling pathway; **(F)** Complement and coagulation cascades.

Subsequently, GO and KEGG analyses were conducted on the above 2,060 DEGs. The findings revealed that DEGs were mainly enriched in functions such as metabolism, extracellular matrix, inflammatory response, and angiogenesis (Figure 4). Figure 4A displays that the GO analysis of all differential genes was mainly enriched in a multicellular organic process, system development, regulation of the multicellular organismal process, and cell periphery. DEGs are primarily enriched in KEGG pathways such as extracellular matrix, tumorigenesis, viral infection, and PI3K-Akt (Figure 4B). Pathview, KEGG visual online analysis software, was utilized to draw a visual mapping pathway diagram of the main signaling pathways. All differential genes were primarily enriched in pathways in cancer (Figure 4C), calcium signaling pathway (Figure 4D), PI3K-Akt signaling pathway (Figure 4E), and complement and coagulation cascades (Figure 4F).

### 3.5 Construction of PPI and screening of core genes

**FIGURE 5:**
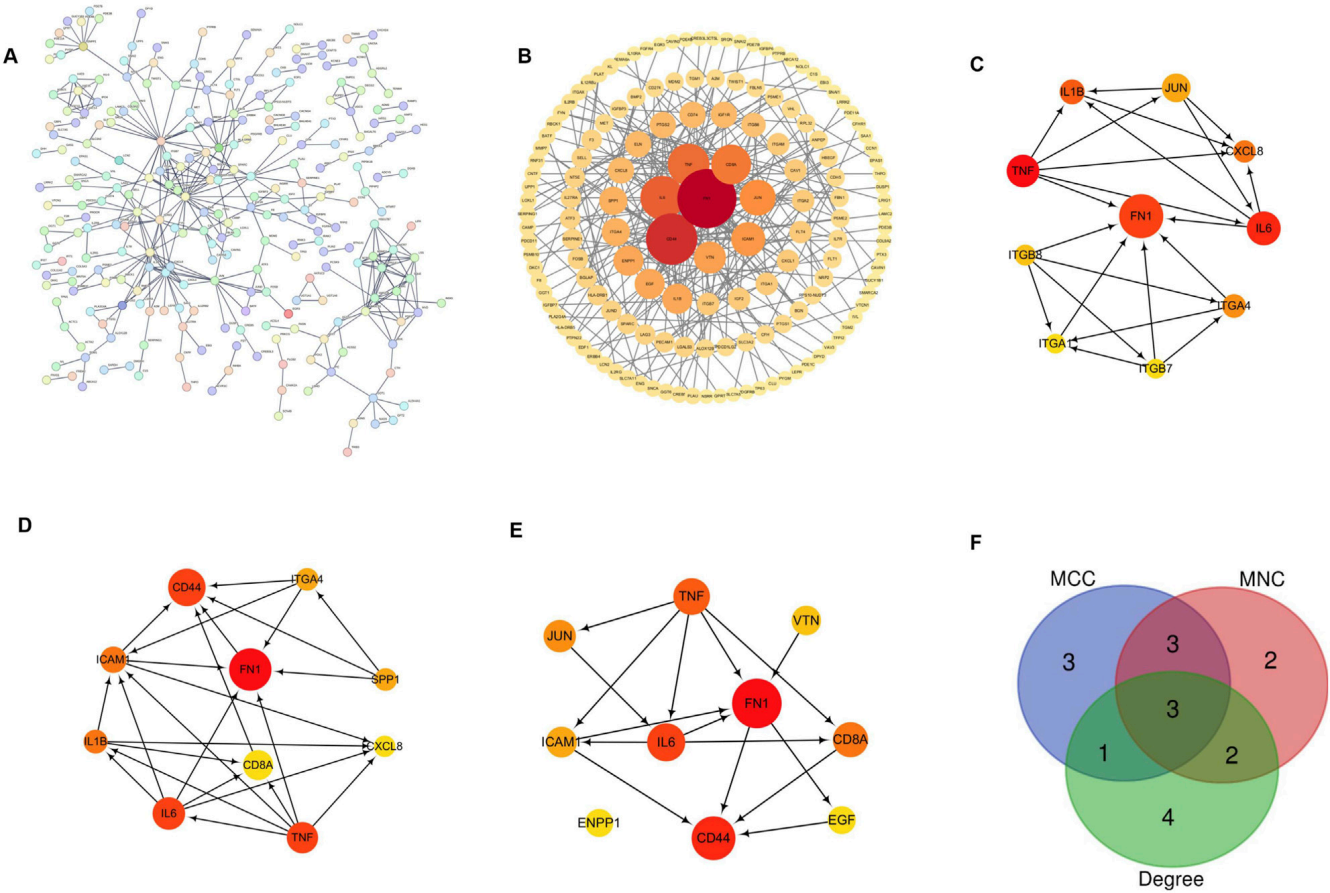
Construction and analysis of protein-protein interaction (PPI) network **(A)** PPI interaction network diagram of differential genes. The circles represent each protein, and the lines represent the interaction between the two proteins; **(B)** The darker the color of the core gene cluster, the higher the score; the larger the shape and the more interaction lines, the higher the score, that is, the closer the interaction between proteins; **(C)** MCC is used to identify the central gene; **(D)** MNC is used to determine the central gene; **(E)** Degree is used to identify the central gene; **(F)** Venn diagram intersection was used to obtain the core genes of TE-1 knockout cell line. The core genes had the strongest correlation between protein interactions after reducing APE1.

The PPI of 2,060 DEGs was constructed from the STRING online database (Figure 5A) and analyzed using Cytoscape software to obtain the core gene cluster (Figure 5B). It was built through the PPI network, ranked according to Hubba nodes, and the top 10 genes were selected using three different algorithms (MCC, MNC, and Degree) to identify core genes (Figures 5C–E). The core genes obtained by the MCC algorithm are as follows: IL-6, ITGB8, CXCL8, ITGA4, FN1, ITGB7, TNF, IL1B, JUN, and ITGA1; those derived by the MNC algorithm: IL-6, SPP1, CXCL8, ITGA4, ICAM1, FN1, TNF, CD44, IL1B, and CD8A; those derived from the Degree algorithm: FCD44, CD8A, EGF, ENPP1, FN1, ICAM1, IL-6, JUN, TNF, and VTN. The darker the color, the higher the score of different algorithms. Subsequently, in the APE1 knockout cell line, the core genes with the strongest protein interaction relationship were obtained through the Venn diagram intersection (Figure 5F). FN1, TNF, and IL-6 are three core genes, among which TNF is an upregulated gene, and FN1 and IL-6 are downregulated genes.

### 3.6 qRT-PCR and western blotting verification of core genes

**FIGURE 6:**
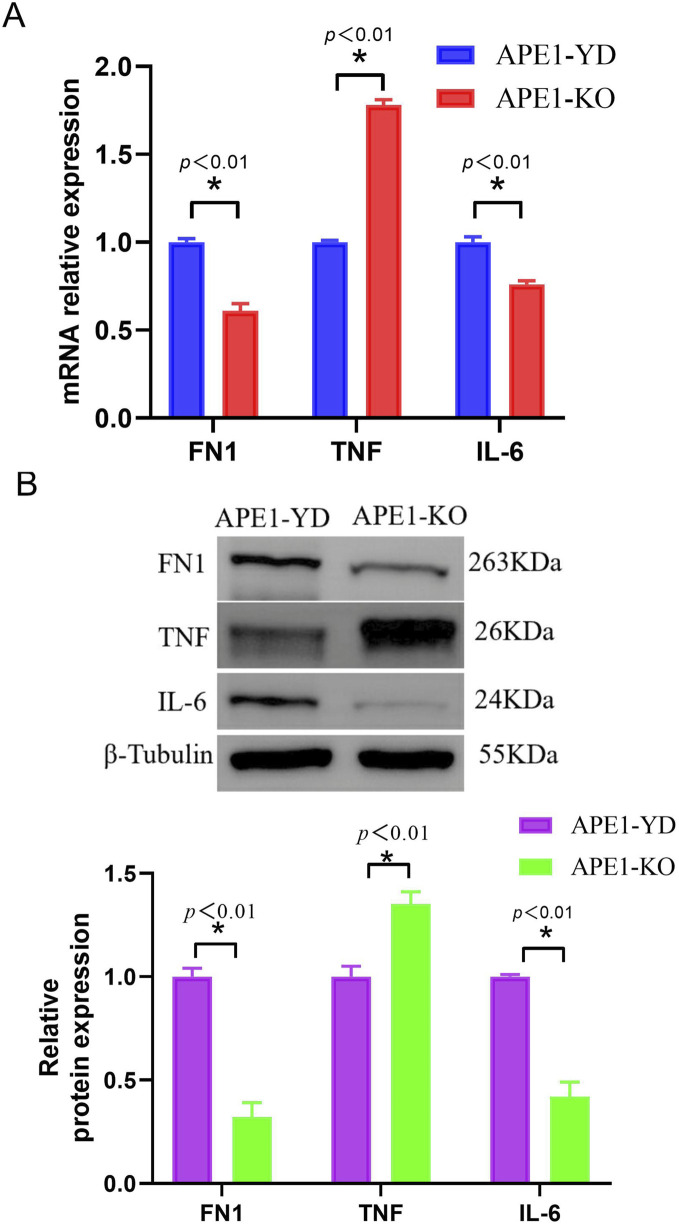
qRT-PCR and Western blot verification of core genes **(A)** Relative expression level of core gene mRNA; **(B)** Relative expression level of core gene protein.

Compared with the sh⁃NC cell line, the mRNA expression levels of TNF in the APE1 knockout group were significantly increased, whereas the mRNA expression levels of FN1 and IL-6 were significantly decreased (Figure 6A), and the differences were statistically significant (P < 0.05). Figure 6B illustrates the Western blotting verification results of the three core genes. Compared with the sh⁃NC cell line, the protein expression levels of TNF in the APE1 knockout group were significantly increased, whereas the protein expression levels of FN1 and IL-6 were significantly decreased (Figure 6B), and the differences were statistically significant (P < 0.05). Experimental results indicate that APE1 knockout will affect the expression of related core genes.

## 4 Discussion

APE1 is a vital DNA repair enzyme that plays a crucial role in maintaining genome integrity and stability (Liu et al., 2021a). Previous studies demonstrated that APE1 also plays an essential role in biological processes, such as cell proliferation, apoptosis, oxidative stress, and inflammation (Liu et al., 2021b). In this research, among the 100 cases of ESCC, we found that APE1 highly expressed in ESCC tissue, and its high expression leads the worse OS. Consequently, we suggest that APE1 is a important factor influencing the prognosis of ESCC. This study applied RNA-seq technology to analyze the esophageal cancer TE-1 cell line with APE1 knockout and explored the differential genes and their functions related to APE1 knockout to gain a deeper understanding of the function and regulatory mechanism of APE1.

Through RNA-seq analysis, differential genes whose expression levels were significantly changed after knocking out the APE1 gene were identified. A total of 2,060 differential genes were screened out, including 1,063 upregulated genes and 997 downregulated genes. Moreover, GO analysis revealed that DEGs were primarily enriched in functions such as metabolism, the extracellular matrix, the inflammatory response, and angiogenesis. Through KEGG analysis, we found that differential genes were mainly enriched in cancer pathways, calcium signaling pathways, PI3K-Akt signaling pathways, and complement and coagulation cascade reaction pathways through KEGG analysis. The above studies conclude that APE1 may primarily affect cell proliferation, survival, metastasis, immune regulation, and tumor immune microenvironment through the above pathways in ESCC cells. A PPI network was constructed to further understand the interactions and regulatory networks between target genes. Through PPI network analysis, we observed multiple interactions between target genes. Finally, among these differential genes, we selected FN1, TNF, and IL-6 as target genes. Among them, TNF is an upregulated gene, and FN1 and IL-6 are downregulated genes.

FN1, an extracellular matrix (ECM) protein linked to cancer metastasis, is upregulated by APE1 to enhance ESCC invasion. Derived from tumor-associated fibroblasts (CAFs), FN1 promotes tumor angiogenesis and ECM stability (To and Midwood, 2011; Amilca-Seba et al., 2021; Biffi and Tuveson, 2021; Kumra et al., 2018; Neve et al., 2014). APE1 knockdown alters actin dynamics through TGF-β1 regulation, enhancing cancer cell motility (Sakai et al., 2015; Tell et al., 2009; Bhakat et al., 2009). FN1 plays a crucial role in the assembly and stability of tumor extracellular matrix, and overexpression of FN1 can promote tumor extracellular matrix formation, thereby enhancing tumor cells' invasion and metastasis ability (Siqueira et al., 2024). On the contrary, FN1 can also regulate the expression and function of APE1. In addition, other studies have reported that FN1 can regulate the expression and activation of APE1 in cervical cancer cells through signaling pathways such as the pyrophosphate tyrosine kinase (FAK) signaling pathway (Zhou et al., 2019).

TNF exhibits antitumor effects through apoptosis induction, but APE1 overexpression counteracts this via NF-κB pathway activation. APE1 inhibitor E3330 reduces TNF-induced IL-6/IL-8 expression, affecting chemotherapy resistance (Park et al., 2016). Prior studies showed that in liver cancer cells, the APE1 inhibitor E3330 prevented the functional activation of the NF-κB pathway by altering APE1 subcellular trafficking and reducing TNF and fatty acid accumulation-induced IL by blocking redox-mediated NF-κB activation (Cesaratto et al., 2013). In endothelial cells, APE1 suppresses TNF-mediated VCAM-1 expression through p38 MAPK inhibition (Kim et al., 2006).

There is a bidirectional interaction between APE1 and IL-6,prior study demostrated that APX3330 inhibits IL-6 production by blocking NF-κB/AP-1 (Oliveira et al., 2022; Mijit et al., 2021), while APE1 overexpression amplifies IL-6 autocrine signaling. IL-6 upregulates APE1 via JAK/STAT3 and enhances its DNA repair capacity through oxidative stress (Johnson et al., 2018; Tang et al., 2022; Nath et al., 2017). Their synergy promotes tumor proliferation, immune evasion, and therapy resistance (Oliveira et al., 2022).

Based on the results of this study and relevant published research, we speculate that APE1 regulates the occurrence and development of esophageal cancer through FN1, TNF, and IL-6, including the following potential mechanisms:1. Direct Transcriptional Regulation Mechanism,APE1 may directly promote the transcription of FN1 and IL-6 by binding to transcription factors such as NF-κB and AP-1. Knockdown of APE1 disrupts this interaction, leading to reduced transcriptional activity and downregulation of FN1 and IL-6. Conversely, the TNF promoter region may contain APE1-dependent inhibitory regulatory elements, and knockout of APE1 relieves this inhibition, upregulating its expression (Sakai et al., 2015; Zhang et al., 2024). 2. Oxidative Stress and NF-κB Pathway. APE1 deficiency impairs DNA damage repair, triggering oxidative stress and activating the NF-κB pathway. Oxidative stress promotes TNF expression by activating NF-κB, while excessive NF-κB activation may indirectly inhibit the transcription of FN1 and IL-6 through competitive binding of transcriptional resources (Oliveira et al., 2022). 3. Extracellular Matrix Remodeling and Tumor Microenvironment. As a core component of the extracellular matrix (ECM), downregulation of FN1 may disrupt tumor cell adhesion to the stroma, inhibiting invasion and metastasis. Meanwhile, upregulation of TNF may recruit immune cells to enhance anti-tumor immunity, while downregulation of IL-6 weakens STAT3-mediated survival signals in tumor cells (Siqueira et al., 2024).

Our research still has some limitations. Further functional studies are warranted to experimentally verify the role of these core genes and deeply explore their regulatory mechanisms by downregulating APE1. For example, gene knockout or overexpression methods can be used to study these core gene methods to explore their impact on the biological behaviors of ESCC cells, such as proliferation, adhesion, migration, and apoptosis, as well as on esophageal cancer genomics and metabolomics implications. Additionally, the interaction between APE1-KO and these genes can be further investigated to find potential regulatory mechanisms. And we will add animal experiments in the future, by constructing a nude mouse model of APE1-KO, and using RT-qPCR, Western blot, immunohistochemistry and other technologies to detect the expression levels of downstream key genes in the two groups of nude mouse tissues. Although this study discovered through KEGG analysis that the differential genes were primarily enriched in pathways such as pathways in cancer, calcium signaling pathway, PI3K-Akt signaling pathway, and complement and coagulation cascades, the discussion section did not analyze these pathways. However, the specific regulatory mechanisms were discussed. Further research can experimentally verify the functions of these pathways in ESCC through *in vitro* or *in vivo* experiments and explore the regulatory mechanism of APE1-KO on these pathways.

In summary, through RNA-seq analysis and functional studies, this study revealed that the knockout of the APE1 gene triggered the differential expression of multiple genes in ESCC cells and discussed the potential functions of these genes in such cells. These findings provide important clues for further studying the mechanism of APE1-KO in developing esophageal cancer and treatment strategies. Future studies can further verify the functions of these DEGs and examine the regulatory network of APE1 and its interactions with other pathways.

## 5 Conclusion

APE1 knockout gene in ESCC cells primarily influences vital pathways associated with metabolism, the extracellular matrix, the inflammatory response, and angiogenesis. APE1 can enhance the transcriptional expressions of FN1 and IL-6 genes while inhibiting the TNF gene. Hence, it is plausible to consider FN1, TNF, and IL-6 as the potential target genes regulated by APE1 in the context of esophageal cancer.

## Data Availability

The datasets presented in this study can be found in online repositories. The names of the repository/repositories and accession number(s) can be found below: https://www.ncbi.nlm.nih.gov/, SAMN45831600, SAMN45831601, SAMN45831602, SAMN45831603, SAMN45831604, SAMN45831605.

## References

[B1] Amilca-Seba K. Sabbah M. Larsen A. K. Denis J. A. (2021). Osteopontin as a regulator of Colorectal cancer progression and its clinical Applications. Cancers (Basel) 13 (15), 3793. 10.3390/cancers13153793 34359694 PMC8345080

[B2] Ando K. Hirao S. Kabe Y. Ogura Y. Sato I. Yamaguchi Y. (2008). A new APE1/Ref-1-dependent pathway leading to reduction of NF-kappaB and AP-1, and activation of their DNA-binding activity. Nucleic Acids Res. 36 (13), 4327–4336. 10.1093/nar/gkn416 18586825 PMC2490748

[B3] Bakman A. S. Ishchenko A. A. Saparbaev M. Fedorova O. S. Kuznetsov N. A. (2022). Pre-steady-state kinetic and mutational insights into mechanisms of endo- and exonuclease DNA processing by mutant forms of human AP endonuclease. Biochim. Biophys. Acta Gen. Subj. 1866 (12), 130198. 10.1016/j.bbagen.2022.130198 35809816

[B4] Bhakat K. K. Mantha A. K. Mitra S. (2009). Transcriptional regulatory functions of mammalian AP-endonuclease (APE1/Ref-1), an essential multifunctional protein. Antioxid. Redox Signal 11 (3), 621–638. 10.1089/ars.2008.2198 18715144 PMC2933571

[B5] Bhat A. A. Lu H. Soutto M. Capobianco A. Rai P. Zaika A. (2018). Exposure of Barrett's and esophageal adenocarcinoma cells to bile acids activates EGFR-STAT3 signaling axis via induction of APE1. Oncogene 37 (46), 6011–6024. 10.1038/s41388-018-0388-8 29991802 PMC6328352

[B6] Biffi G. Tuveson D. A. (2021). Diversity and Biology of cancer-associated fibroblasts. Physiol. Rev. 101 (1), 147–176. 10.1152/physrev.00048.2019 32466724 PMC7864232

[B7] Caston R. A. Gampala S. Armstrong L. Messmann R. A. Fishel M. L. Kelley M. R. (2021). The multifunctional APE1 DNA repair-redox signaling protein as a drug target in human disease. Drug Discov. Today 26 (1), 218–228. 10.1016/j.drudis.2020.10.015 33148489 PMC7855940

[B8] Cesaratto L. Codarin E. Vascotto C. Leonardi A. Kelley M. R. Tiribelli C. (2013). Specific inhibition of the redox activity of ape1/ref-1 by e3330 blocks tnf-α-induced activation of IL-8 production in liver cancer cell lines. PLoS One 8 (8), e70909. 10.1371/journal.pone.0070909 23967134 PMC3744539

[B9] Gampala S. Shah F. Zhang C. Rhodes S. D. Babb O. Grimard M. (2021). Exploring transcriptional regulators Ref-1 and STAT3 as therapeutic targets in malignant peripheral nerve sheath tumours. Br. J. Cancer 124 (9), 1566–1580. 10.1038/s41416-021-01270-8 33658640 PMC8076291

[B10] Gros L. Ishchenko A. A. Ide H. Elder R. H. Saparbaev M. K. (2004). The major human AP endonuclease (Ape1) is involved in the nucleotide incision repair pathway. Nucleic Acids Res. 32 (1), 73–81. 10.1093/nar/gkh165 14704345 PMC373275

[B11] Hong J. Y. Oh H. H. Park S. Y. Park Y. L. Cho S. B. Joo Y. E. (2023). Expression of apurinic/apyrimidinic endonuclease 1 in Colorectal cancer and its relation to tumor progression and prognosis. In Vivo 37 (5), 2070–2077. 10.21873/invivo.13304 37652525 PMC10500501

[B12] Johnson D. E. O'Keefe R. A. Grandis J. R. (2018). Targeting the IL-6/JAK/STAT3 signalling axis in cancer. Nat. Rev. Clin. Oncol. 15 (4), 234–248. 10.1038/nrclinonc.2018.8 29405201 PMC5858971

[B42] Kanehisa M. Araki M. Goto S. Hattori M. Hirakawa M. Itoh M. (2008). KEGG for linking genomes to life and the environment. Nucleic Acids Research, 36 (Database issue), D480–D484. 10.1093/nar/gkm882 18077471 PMC2238879

[B13] Kim C. S. Son S. J. Kim E. K. Kim S. N. Yoo D. G. Kim H. S. (2006). Apurinic/apyrimidinic endonuclease1/redox factor-1 inhibits monocyte adhesion in endothelial cells. Cardiovasc Res. 69 (2), 520–526. 10.1016/j.cardiores.2005.10.014 16325162

[B14] Kumar S. Zhao J. Talluri S. Buon L. Mu S. Potluri L. B. (2023). Elevated APE1 Dysregulates homologous recombination and cell cycle driving genomic evolution, tumorigenesis, and chemoresistance in esophageal adenocarcinoma. Gastroenterology 165 (2), 357–373. 10.1053/j.gastro.2023.04.035 37178737 PMC10524563

[B15] Kumra H. Sabatier L. Hassan A. Sakai T. Mosher D. F. Brinckmann J. (2018). Roles of fibronectin isoforms in neonatal vascular development and matrix integrity. PLoS Biol. 16 (7), e2004812. 10.1371/journal.pbio.2004812 30036393 PMC6072322

[B16] Li Q. Zhou Z. W. Duan W. Qian C. Y. Wang S. N. Deng M. S. (2021). Inhibiting the redox function of APE1 suppresses cervical cancer metastasis via disengagement of ZEB1 from E-cadherin in EMT. J. Exp. Clin. Cancer Res. 40 (1), 220. 10.1186/s13046-021-02006-5 34210327 PMC8246661

[B17] Liu T. C. Guo K. W. Chu J. W. Hsiao Y. Y. (2021a). Understanding APE1 cellular functions by the structural preference of exonuclease activities. Comput. Struct. Biotechnol. J. 19, 3682–3691. 10.1016/j.csbj.2021.06.036 34285771 PMC8258793

[B18] Liu T. C. Lin C. T. Chang K. C. Guo K. W. Wang S. Chu J. W. (2021b). APE1 distinguishes DNA substrates in exonucleolytic cleavage by induced space-filling. Nat. Commun. 12 (1), 601. 10.1038/s41467-020-20853-2 33504804 PMC7841161

[B19] Logsdon D. P. Grimard M. Luo M. Shahda S. Jiang Y. Tong Y. (2016). Regulation of HIF1α under Hypoxia by APE1/ref-1 impacts CA9 expression: dual targeting in Patient-derived 3D Pancreatic cancer models. Mol. Cancer Ther. 15 (11), 2722–2732. 10.1158/1535-7163.mct-16-0253 27535970 PMC5097013

[B20] Long K. Gu L. Li L. Zhang Z. Li E. Zhang Y. (2021). Small-molecule inhibition of APE1 induces apoptosis, pyroptosis, and necroptosis in non-small cell lung cancer. Cell Death Dis. 12 (6), 503. 10.1038/s41419-021-03804-7 34006852 PMC8131371

[B21] Lu H. Cao L. L. Ballout F. Belkhiri A. Peng D. Chen L. (2023). Reflux conditions induce E-cadherin cleavage and EMT via APE1 redox function in oesophageal adenocarcinoma. Gut 73 (1), 47–62. 10.1136/gutjnl-2023-329455 37734913 PMC10872865

[B22] Lu X. Zhao H. Yuan H. Chu Y. Zhu X. (2021). High nuclear expression of APE1 correlates with unfavorable prognosis and promotes tumor growth in hepatocellular carcinoma. J. Mol. Histol. 52 (2), 219–231. 10.1007/s10735-020-09939-9 33392892

[B23] Malfatti M. C. Antoniali G. Codrich M. Tell G. (2021). Coping with RNA damage with a focus on APE1, a BER enzyme at the crossroad between DNA damage repair and RNA processing/decay. DNA Repair (Amst). 104, 103133. 10.1016/j.dnarep.2021.103133 34049077

[B24] McIlwain D. W. Fishel M. L. Boos A. Kelley M. R. Jerde T. J. (2018). APE1/Ref-1 redox-specific inhibition decreases survivin protein levels and induces cell cycle arrest in prostate cancer cells. Oncotarget 9 (13), 10962–10977. 10.18632/oncotarget.23493 29541389 PMC5834255

[B25] Mijit M. Caston R. Gampala S. Fishel M. L. Fehrenbacher J. Kelley M. R. (2021). APE1/Ref-1 - One target with multiple Indications: emerging Aspects and new directions. J. Cell Signal 2 (3), 151–161. 34557865 PMC8457357

[B26] Nath S. Roychoudhury S. Kling M. J. Song H. Biswas P. Shukla A. (2017). The extracellular role of DNA damage repair protein APE1 in regulation of IL-6 expression. Cell Signal 39, 18–31. 10.1016/j.cellsig.2017.07.019 28751279 PMC5592147

[B27] Neve A. Cantatore F. P. Maruotti N. Corrado A. Ribatti D. (2014). Extracellular matrix modulates angiogenesis in physiological and pathological conditions. Biomed. Res. Int. 2014, 756078. 10.1155/2014/756078 24949467 PMC4052469

[B28] Oliveira T. T. Coutinho L. G. de Oliveira L. O. A. Timoteo A. R. S. Farias G. C. Agnez-Lima L. F. (2022). APE1/Ref-1 role in inflammation and immune response. Front. Immunol. 13, 793096. 10.3389/fimmu.2022.793096 35296074 PMC8918667

[B29] Park M. S. Choi S. Lee Y. R. Joo H. K. Kang G. Kim C. S. (2016). Secreted APE1/Ref-1 inhibits TNF-α-stimulated endothelial inflammation via thiol-disulfide exchange in TNF receptor. Sci. Rep. 6, 23015. 10.1038/srep23015 26964514 PMC4786854

[B30] Sakai Y. Yamamori T. Yasui H. Inanami O. (2015). Downregulation of the DNA repair enzyme apurinic/apyrimidinic endonuclease 1 stimulates transforming growth factor-β1 production and promotes actin rearrangement. Biochem. Biophys. Res. Commun. 461 (1), 35–41. 10.1016/j.bbrc.2015.03.163 25858321

[B31] Siqueira P. B. de Sousa Rodrigues M. M. de Amorim Í. S. S. da Silva T. G. da Silva Oliveira M. Rodrigues J. A. (2024). The APE1/REF-1 and the hallmarks of cancer. Mol. Biol. Rep. 51 (1), 47. 10.1007/s11033-023-08946-9 38165468

[B32] Sriramajayam K. Peng D. Lu H. Zhou S. Bhat N. McDonald O. G. (2021). Activation of NRF2 by APE1/REF1 is redox-dependent in Barrett's related esophageal adenocarcinoma cells. Redox Biol. 43, 101970. 10.1016/j.redox.2021.101970 33887608 PMC8082268

[B33] Sung H. Ferlay J. Siegel R. L. Laversanne M. Soerjomataram I. Jemal A. (2021). Global cancer statistics 2020: GLOBOCAN estimates of incidence and mortality worldwide for 36 cancers in 185 countries. CA Cancer J. Clin. 71, 209–249. 10.3322/caac.21660 33538338

[B34] Tang C. H. Qin L. Gao Y. C. Chen T. Y. Xu K. Liu T. (2022). APE1 shRNA-loaded cancer stem cell-derived extracellular vesicles reverse Erlotinib resistance in non-small cell lung cancer via the IL-6/STAT3 signalling. Clin. Transl. Med. 12 (5), e876. 10.1002/ctm2.876 35605028 PMC9126360

[B35] Tell G. Quadrifoglio F. Tiribelli C. Kelley M. R. (2009). The many functions of APE1/Ref-1: not only a DNA repair enzyme. Antioxid. Redox Signal 11 (3), 601–620. 10.1089/ars.2008.2194 18976116 PMC2811080

[B36] To W. S. Midwood K. S. (2011). Plasma and cellular fibronectin: distinct and independent functions during tissue repair. Fibrogenes. Tissue Repair 4, 21. 10.1186/1755-1536-4-21 PMC3182887 21923916

[B37] Wen X. Lu R. Xie S. Zheng H. Wang H. Wang Y. (2016). APE1 overexpression promotes the progression of ovarian cancer and serves as a potential therapeutic target. Cancer Biomark. 17 (3), 313–322. 10.3233/cbm-160643 27802207 PMC13020497

[B38] Zhang Y. Wang J. (2010). Anticancer clinical utility of the apurinic/apyrimidinic endonuclease/redox factor-1 (APE/Ref-1). Chin. J. Cancer 29 (3), 333–339. 10.5732/cjc.009.10285 20193121

[B39] Zhang Z. Lin Y. Pan X. Chen S. (2024). Inhibition of non-small cell lung cancer metastasis by knocking down APE1 through regulating myeloid-derived suppressor cells-induced immune disorders. Aging (Albany NY) 16 (12), 10435–10445. 10.18632/aging.205938 38885059 PMC11236315

[B40] Zhou Y. Shu C. Huang Y. (2019). Fibronectin promotes cervical cancer tumorigenesis through activating FAK signaling pathway. J. Cell Biochem. 120 (7), 10988–10997. 10.1002/jcb.28282 30977220

[B41] Zhu H. Ma X. Ye T. Wang H. Wang Z. Liu Q. (2023). Esophageal cancer in China: Practice and research in the new era. Int. J. Cancer 152 (9), 1741–1751. 10.1002/ijc.34301 36151861

